# Low fluoroscopy permanent His bundle pacing using a new electroanatomic mapping system (KODEX EPD). A multicenter experience

**DOI:** 10.1002/joa3.12803

**Published:** 2022-12-30

**Authors:** Antonio Scarà, Paolo Golia, Domenico Grieco, Alessio Borrelli, Ermenegildo De Ruvo, Edoardo Bressi, Alessandro Politano, Lucia De Luca, Giuseppe Bruni, Alessandro Fagagnini, Marco Panuccio, Marco Rebecchi, Paolo Zecchi, Francesco Solimene, Leonardo Calò, Luigi Sciarra

**Affiliations:** ^1^ Policlinico Casilino Hospital Rome Italy; ^2^ NS della Mercede Hospital Rome Italy; ^3^ Montevergine Hospital Avellino Italy

**Keywords:** electroanatomical mapping system, fluoroscopy exposure reduction, His bundle pacing, physiological pacing

## Abstract

**Background:**

His bundle pacing (HBP) may be a challenging procedure, often involving a long fluoroscopic time (FT) and a long procedural time (PT). We sought to evaluate whether the use of a new nonfluroscopic mapping (NFM) system, the KODEX‐EPD, is able to reduce FT and PT when mapping is performed by the pacing catheter rather than an electrophysiological mapping catheter.

**Methods and Results:**

We included 46 consecutive patients (77 ± 8 years; 63% male) who underwent HBP; in 22 a NFM‐guided procedure with the KODEX‐EPD system was performed (group 1), whereas in 24 a conventional fluoroscopy‐guided approach was used (group 2). Pacing indications were sick sinus syndrome in 13, atrioventricular block in 21, and cardiac resynchronization therapy in 12 cases. Both a lumen‐less fixed helix lead and a stylet‐driven extendable helix lead were used, respectively, in 24% and 76% of patients. HBP was successful in 22 patients (100%) in group 1 and 23 patients (96%) in group 2. The FT was significantly reduced in group 1 (183 ± 117 s vs 464.1 ± 352 s in group 2, *p* = .012). There were no significant differences between groups in PT and other procedural outcomes.

**Conclusions:**

The KODEX‐EPD system may be safely used in HBP procedures. It is effective in reducing ionizing radiation exposure, as evidenced by the significant drop in FT, without increasing PT.

## Graphical abstract

His bundle pacing (HBP) may be a challenging procedure, often involving a long fluoroscopic time (FT) and a long procedural time (PT). The KODEX‐EPD system may be safely used in HBP procedures. It is effective in reducing ionizing radiation exposure, as evidenced by the significant drop in FT, without increasing PT.

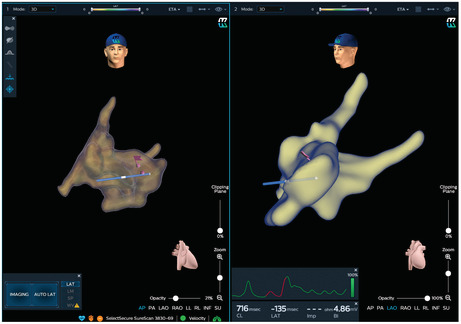

## 1 BACKGROUND

Permanent His bundle pacing (HBP) has been proposed, many years ago, as the more physiological way to pace the ventricles.^1^, ^2^ In fact, in the absence of ventricular conduction disturbances, depolarization of the ventricles through the His‐Purkinje system preserves normal synchronous ventricular activation, avoiding the dyssynchrony induced by right ventricular pacing.^3^ In patients undergoing pacemaker implantation in whom an elevated percentage of paced beats is expected, HBP may therefore prevent the onset of pacing‐induced cardiomyopathy.^4^, ^5^ Moreover, in many cases, HBP may correct bundle branch blocks, by pacing slightly distally to the site of the conduction block.^6^ However, despite being the most desirable form of pacing, HBP presents some critical issues that have prevented its widespread diffusion. Most of these issues are related to the procedure itself, as it is often difficult to find a proper His bundle site with a reasonable capture threshold, in a reasonable time. As a result, although largely dependent on the operator's experience, the average fluoroscopic time (FT) in HBP is usually higher than in conventional pacing procedures, often rising above 10 min^5^, ^7^; for the same reasons, procedural time (PT), which usually express the overall complexity of the procedure, is also greater in HBP than in right ventricular pacing.^8^

The former is of particular concern because ionizing radiations are proven to cause adverse biological effects in both exposed patients and medical staff.^9^ Therefore, any practical skill or technological advance able to reduce FT and, possibly, PT, can be useful in increasing the long‐term overall safety of physiological pacing as well as its worldwide spreading.

The KODEX‐EPD system (EPD Solutions, Philips) is a relatively new tridimensional (3D) system of nonfluoroscopic mapping (NFM). This system creates computed tomography‐like 3D images of cardiac anatomy by exploiting the specific dielectric properties of biological tissues; as cardiac structures induce gradients in the electrical field transmitted and detected from the exterior skin patches and the roving intracardiac catheters, information can be acquired and used by the system to calculate the geometric characteristics of the 3D image.^10^

The aim of the present study was to assess the feasibility and safety of performing HBP using the KODEX‐EPD system and to compare outcomes with a group of patients undergoing conventional fluoroscopy‐guided HBP.

## 2 METHODS

### 2.1 Population of the study

This is a prospective observational multicenter study, conducted between July and December 2021, among three different centers (Policlinico Casilino 14 cases—18 controls; NS della Mercede Hospital six cases—four controls; Montevergine Hospital two cases—two controls).

Permanent HBP was attempted in all patients >18 years of age with an indication for permanent pacemaker implantation, based on current guidelines^11^; patients with an indication for cardiac resynchronization therapy (CRT) and complete left bundle branch block, according to Strauss criteria,^12^ were also included. Twenty‐two patients were assigned to undergo KODEX‐guided low fluoroscopy implantation (group 1). They were compared with 24 patients who underwent HBP using conventional mapping under fluoroscopic guidance (group 2).

All the procedures were performed with the use of a single‐arm fluoroscopy. The allocation to each group was not randomized but determined by organization and scheduling issues of the electrophysiology laboratory; no patient was preselected for the use of NFM on the basis of additional clinical criteria beyond the conventional indication of pacemaker implantation.

### 2.2 Procedure description

#### 2.2.1 Standard fluoroscopy‐guided HBP protocol

Conventional HBP was performed under fluoroscopic guidance using lumen‐less fixed helix leads (Select Secure lead, model 3830, 69 cm, Medtronic Inc.) or stylet‐driven extendable helix leads (Solia S 60 cm, Biotronik), as previously described.^3^, ^13^ The lead was delivered through a fixed curve sheath (C315HIS, Medtronic Inc., or Selectra 3D, Biotronik). His bundle electrograms were mapped with the pacing lead in a unipolar fashion and recorded by the Medtronic pacing system analyzer (model 2290) or by an electrophysiological recording system (91.6% of overall cases), based on the operator preference. The region of interest was carefully mapped until pacing resulted in His bundle capture. The lead was then fixed at the chosen endocardial site by clockwise rotations.

The His bundle capture thresholds and bundle branch correction thresholds were assessed and recorded at a pulse width of 1.0 ms. When HBP with an acceptable threshold (<1.5 V@1 ms) could not be achieved after a His lead‐fluoroscopy time of 20 min, it was considered a failure. Although these procedures were performed by using fluoroscopy, operators tried to minimize its use. When CRT was required, correction of the left bundle branch block (LBBB) was a procedural endpoint.

#### 2.2.2 KODEX guided HBP protocol

NFM was an essential part of the procedure. The KODEX‐EPD system was used for mapping and catheter navigation.

The pacing lead and the delivery sheath were introduced through the cephalic or axillar approach; the pacing lead was then connected to the navigation system using a sterilized cable with alligator clips (SJM Threshold cable, Number 401718, Abbott).

The operator attempted to rely on NFM for lead navigation rather than fluoroscopy.

The pacing lead was manipulated in the septal region, in order to build a local tridimensional map and to localize His bundle signal area, through live visualization of the recorded electrograms in a bipolar or unipolar mode; moreover, pacing from the lead and tagging of selective or nonselective His bundle capture sites^14^ were recorded.

After mapping and confirming the site with successful His bundle capture, the lead was finally fixed by clockwise rotations. When paced‐QRS morphology suggested an exclusive right ventricular septal pacing, without any conduction fibers involvement it was not considered as nonselective HBP, a new attempt was performed and the pacing lead was navigated to another target site based on the previous mapping.

When CRT was required, correction of LBBB was a procedural endpoint, as well as for standard procedures.

### 2.3 Data collection and definitions

We collected demographic data, procedural indications, and data as procedural success, evidence of selective or nonselective His bundle capture, PT, FT. PT and FT were defined as the total duration respectively of the procedure and fluoroscopy exposure. Procedural success was defined as the ability to implant the lead at the His bundle region with electrocardiographic evidence of HBP. Selective or nonselective HBP was determined based on previously published criteria.^14^ Briefly, selective HBP was defined as ventricular activation occurring exclusively via the His‐Purkinje system, as evidenced by a paced QRS identical to baseline (in patients presenting with a normal QRS), possibly associated with a significantly narrower paced QRS in patients presenting with a bundle branch block.

Nonselective HBP was defined based on the capture of the basal ventricular septum in addition to His bundle capture; in these patients, usually, there was no isoelectric interval between the pacing stimulus and QRS onset and QRS morphology was slightly different from baseline even in the absence of a pre‐existing bundle branch block.

### 2.4 Statistical analysis

Continuous variables were reported as mean ± SD. Comparisons were made using the *χ* ^2^ test for categorical variables and the Pearson correlation coefficient for continuous variables. The Spearman correlation coefficient was used for nonparametric data. *p* ≤ .05 was considered statistically significant.

## 3 RESULTS

The clinical and demographic characteristics of the two groups are reported in Table 1. No significant differences were observed at baseline between the groups. Pacing indications were also similar in the two groups: a total of 13 patients (32% in group 1 and 25% in group 2, *p* = .608) presented with sick sinus syndrome, 21 patients (45% in group 1 and 46% in group 2, *p* = .979) presented with atrioventricular block: in particular 10 patients had a Mobitz 1 second degree block (5 in group 1 and 5 in group 2), five patients suffered from Mobitz 2 second degree block (2 in group 1 and 3 in group 2), six patients had a third‐degree AV block (3 in group 1 and 3 in group 2).

**TABLE 1:** The table shows the clinical characteristics of the study and control population

	Total (*n* = 46)	HBP‐Kodex (*n* = 22)	HBP‐EGM (*n* = 24)	*p* value
Age (years)	77 ± 8	79 ± 10	74 ± 8	.482
Male, *n* (%)	29 (63)	15 (68)	14 (58)	.489
Body weight (kg)	75.9 ± 8.8	76.5 ± 8.9	75.4 ± 8.9	.683
BMI (kg/m^2^)	26 ± 2	25 ± 2	26 ± 2	.619
LVEF (%)	52 ± 14	54 ± 14	51 ± 13	.921
Hypertension (%)	20 (43)	10 (45)	10 (42)	1.000
Ischaemic heart disease (%)	9 (19)	4 (18)	5 (20)	1.000
Dilated cardiomyopathy (%)	12 (26)	5 (23)	7 (29)	.619
Pacing indication				
SSS, *n* (%)	13 (28)	7 (32)	6 (25)	.608
AVB, *n* (%)	21 (46)	10 (45)	11 (46)	.980
2nd degree AVB	5 (11)	2 (9)	3 (12)	
3rd degree AVB	6 (13)	3 (14)	3 (12)	
CRT, *n* (%)	12 (26)	5 (23)	7 (29)	.619
HV interval, ms	55.7 ± 13.0	55.6 ± 11.3	55.7 ± 14.5	.979
QRS spontaneous	117 ± 30	113 ± 28	119 ± 31	.501
QRS paced	106 ± 17	102 ± 16	110 ± 18	.468

*Note*: Values are shown as mean ± standard deviation and percentage (%) is also reported.

Abbreviations: AVB, atrioventricular block; BMI, body mass index; CRT, cardiac resynchronization therapy; LVEF, left ventricular ejection fraction; SSS, means sick sinus syndrome.

Finally, 12 patients (23% in group 1 and 29% in group 2, *p* = .619) had CRT indications.

Mean HV interval, mean spontaneous QRS, and mean stimulated QRS durations were also collected.

**FIGURE 1:**
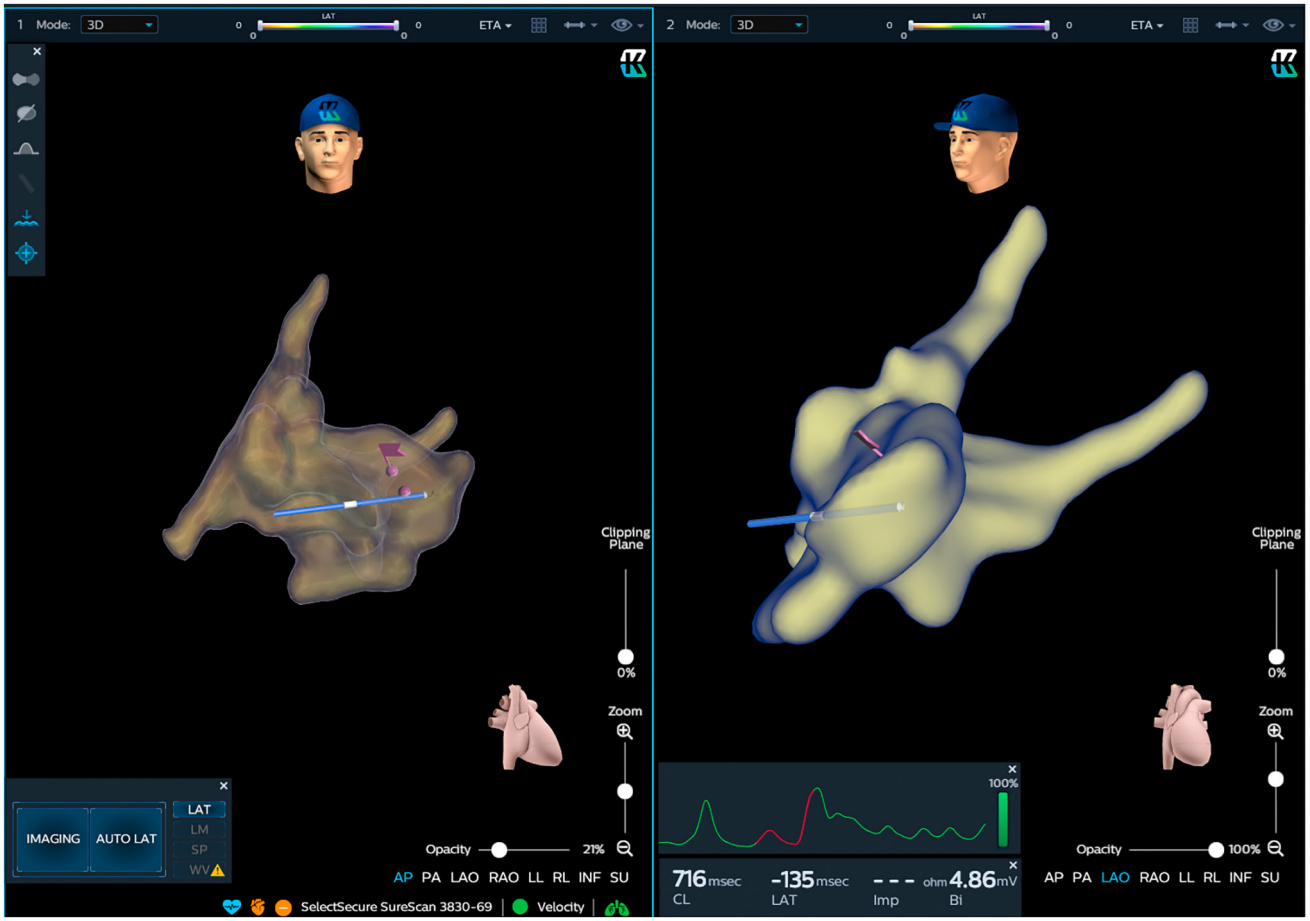
Anatomical map with His lead using KODEX‐EPD system: Shows a case of His lead implantation using the Kodex system. Pacing catheter placement on His site has been visualized in the right anterior oblique (RAO projection (left side of picture) and in the left anterior oblique (LAO) projection (right side of picture). The violet flag indicates a selective HBP site, whereas the violet dot identifies a nonselective one. In this case, a nonselective HBP site was selected because of threshold reason.

In group 1 the electroanatomical map was directly acquired using the HBP lead (Figure 1).

**FIGURE 2:**
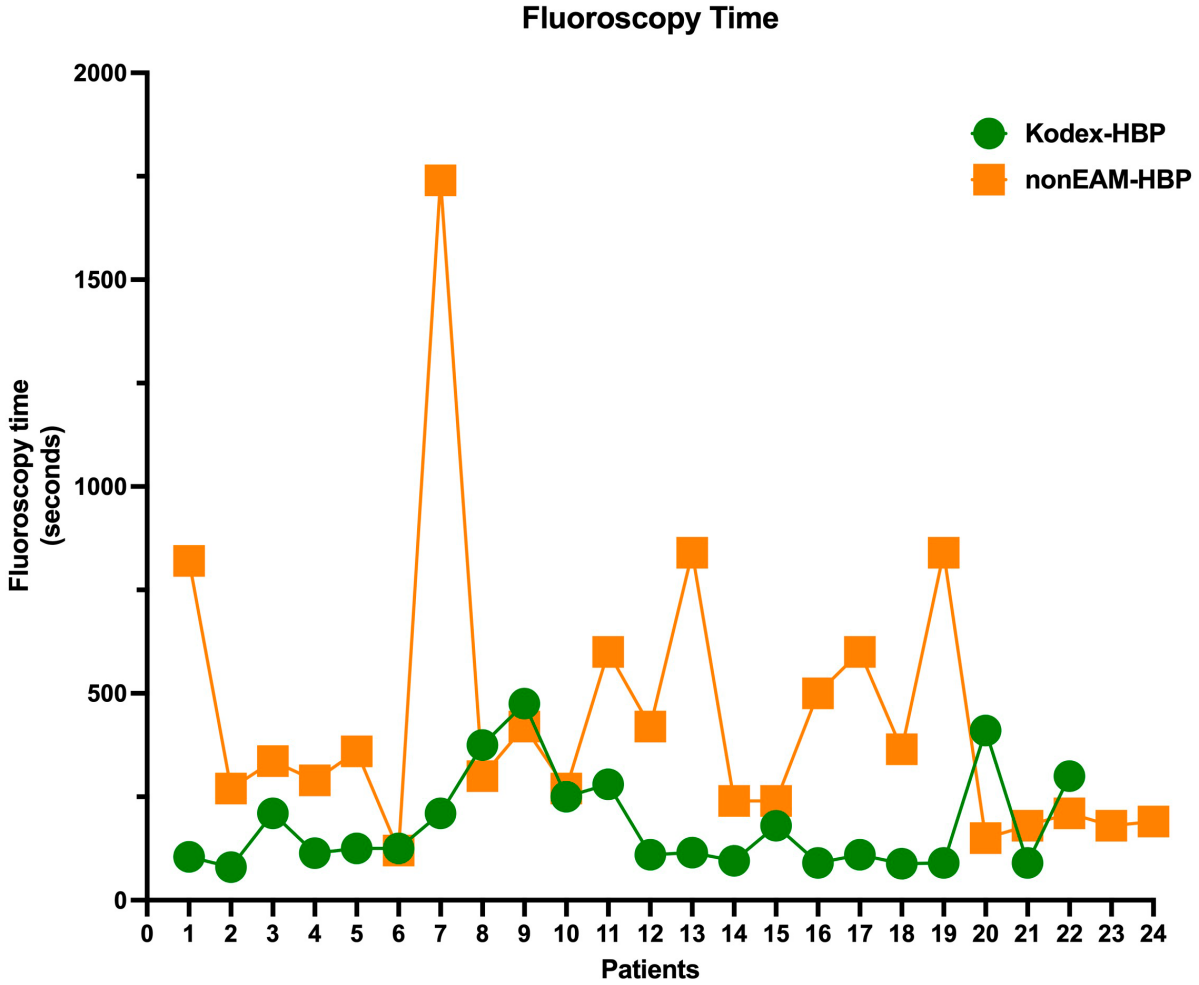
Distribution of fluoroscopy time among the entire population study: Orange squares identify each patient undergoing standard fluoroscopy procedure. Green circles represent each patient undergoing a Kodex‐guided procedure.

Collected procedural data (Table 2) showed a mean FT of 183 ± 117 s in group 1 and of 464 ± 352 s in group 2, which was a statistically significant difference (*p* = .012). The distribution of FT among the entire population study has been reported (Figure 2).

**TABLE 2:** Procedural parameters

	Kodex‐HBP	Fluoro‐HBP	*p* value
HBP threshold (V)^a^	0.8 ± 0.2	0.8 ± 0.3	.156
Impedance (ohms)	793 ± 166	776 ± 178	.892
R wave amplitude (mV)	4.5 ± 2.9	4.3 ± 2.7	.809
Fluoro time (s)	183 ± 117	464 ± 352	**.012**
Procedural time (min)	73 ± 15	76 ± 17	.469
S‐HBP, *n* (%)	11(50)	11(46)	.777
NS‐HBP, *n* (%)	11(50)	12(50)	1.000
Failure, *n* (%)	0(0)	1(4)	.333
HB catheter, *n* (%)			
Medtronic3830 *n* (%)	5(23)	6(25)	.857
BiotronikSolia *n* (%)	17(77)	18(75)	.857

*Note*: Values are shown as mean ± standard deviation and percentage (%) is also reported. Bold indicates the only statistically significant *p* value.

Abbreviations: NS‐HBP, nonselective HIS bundle pacing; S‐HBP, selective HIS bundle pacing.

^a^ Duration 1 ms.

Mean PT was similar in the two groups (73 ± 15 versus 76 ± 17 min), without a statistical significance (*p* = .469).

**FIGURE 3:**
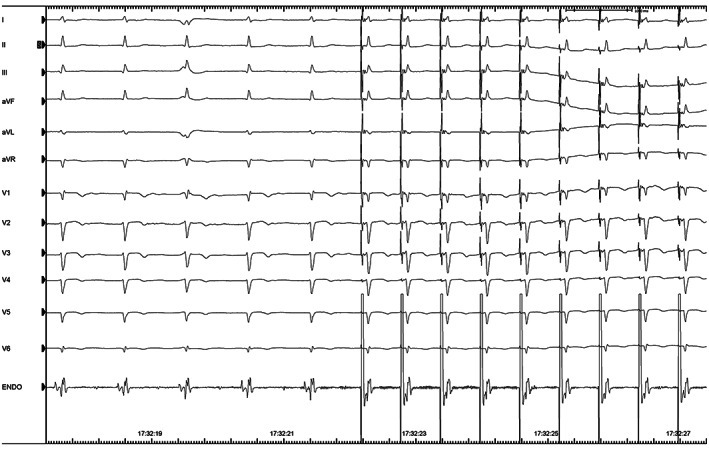
Selective capture of His bundle: An example of selective His bundle pacing. After five spontaneous ventricular activations, selective His bundle stimulation has been obtained (QRS maintain the same shape, and a delay between stimulus and QRS can be observed).

Selective HBP (Figure 3) was achieved in 48% of patients (11 in group 1 and 11 in group 2, *p* = .777), and nonselective HBP in 50% (11 in group 1 and 12 in group 2, *p* = 1.000). One failure was reported in group 2: it was a case of complete AV block with wide QRS, in which wasn't possible to find a His signal; in this case, a simple ventricular septal stimulation was chosen.

**FIGURE 4:**
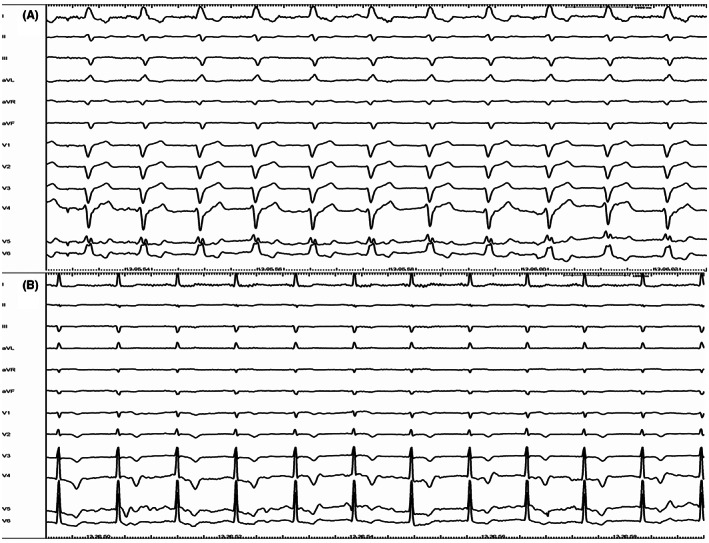
Correction of CLBBB: Panel A shows a basal ECG with CLBBB. Panel B represents the ECG from the same patient at the end of procedure. CLBBB has been completely corrected by a selective HBP.

Correction on LBBB was achieved in all patients receiving CRT (Figure 4).

In 24% of patients (five in group 1 and 6 in group 2, *p* = .857) Medtronic 3830 lead was used, while Biotronik Solia lead was used in 76% of patients (17 in group 1 and 18 in group 2, *p* = .857).

No acute procedural or in‐hospital complications were observed in both group.

### 3.1 Follow‐up

Patients were evaluated at 1 and 6 months. An increase of capture threshold ≥1 V from baseline was observed in 3 patients in group 1 (13.6%) and 4 patients in group 2 (17.4%) with no statistical difference (*p* = .949) at 6 months follow‐up. No lead dislodgements were observed, and no patients required lead revision during follow‐up (Table 3).

**TABLE 3:** 1 and 6 months follow‐up

	1 month follow‐up		*p* value	6 months follow‐up		*p* value
	Kodex‐HBP	Fluoro‐ HBP		Kodex‐HBP	Fluoro‐HBP	
HBP threshold (V)^a^	0.9 ± 0.4	1.0 ± 0.6	0.513	1.3 ± 0.5	1.5 ± 0.5	.182
Lead dislodgment, (*n*%)	0 (0)	0 (0)	—	0 (0)	0 (0)	—
Lead revision, (*n*%)	0 (0)	0 (0)	—	0 (0)	0 (0)	—

*Note*: Values are shown as mean ± standard deviation.

^a^ Duration 1 ms.

## 4 DISCUSSION

The results of our study confirm the feasibility and safety of utilizing the novel KODEX‐EPD system in HBP procedures (Figure 3), and the possibility of obtaining, with the use of this system, a marked drop in procedure‐related ionizing radiations exposure for both patient and medical staff, as evidenced by the significant reduction of FT.

Since its original description in 1967^1^ and its first clinical application in patients undergoing atrioventricular nodal ablation by Deshmukh et al in 2000,^2^ HBP has been considered the optimal form of pacing, because of its ability to maintain normal activation sequence of the ventricles in subjects without ventricular conduction defects, thus preventing dyssynchrony and its long‐term consequences. On the contrary, in subjects with ventricular conduction defects, HBP has revealed the ability to normalize QRS, by pacing distally to the site of block in a high percentage of cases. However, it was only in more recent times, with the use of improved pacing leads and delivery sheaths that HBP gained more widespread acceptance.^3^, ^8^ Left bundle branch area pacing can be considered in some respects an evolution of HBP, as it produces an activation sequence that is only slightly less physiological than HBP, but with a greater rate of correction of the left bundle branch block; importantly, pacing parameters are better in the short and long term with this new technique.^15^ However, at present, the biggest problem with physiological pacing, possibly preventing many centers from undertaking it is the greater complexity of the procedure in comparison with usual pacing approaches, which is particularly feared by implanters who lack interventional electrophysiology expertise. As a reflection of this complexity, in a direct comparison between HBP and right ventricular pacing, the former was associated with significantly longer fluoroscopy and procedure duration.^5^, ^8^ Particular concern exists about the high dose of fluoroscopy normally needed for HBP; FT is typically slightly above 10 min in a HBP procedure,^5^, ^7^, ^8^ but it can be much more when a challenging anatomy is encountered.^16^ Exposure to ionizing radiations is associated with DNA damage (mainly by the formation of hydroxyl radicals from water molecules), which carries harm to human health by both a stochastic and deterministic mechanism of action, with a consequent nonnegligible risk of malignancy and other diseases. Particularly in susceptible categories of patients, such as children and pregnant women, ionizing radiation exposure should be reduced as much as possible. Although traditional protection tools such as lead aprons, thyroid collars, lead glasses, and more recently improved X‐ray systems have proven to be effective measures, the need to reduce ionizing radiation exposure for both medical staff and patients still remains. Zanon et al recently demonstrated that in HBP procedures an electrophysiological mapping‐based approach directed to electrograms of the His bundle area is able to greatly reduce FT.^17^ However, the prolonged FT required to achieve HBP, often presenting at the beginning of the operator's learning curve or in anatomically challenging cases remains one of the main issues of this procedure.

NFM systems have been recently introduced in interventional electrophysiology, which significantly reduces the need for the fluoroscopic visualization of catheters, and, therefore, also radiological exposure for patients and operators. In addition, the 3D anatomical image provided by NFM is more efficient in guiding the catheter movements compared with the two‐dimensional fluoroscopic image, and in many cases can facilitate complex arrhythmias ablation.

NFM has also been successfully utilized for implantation procedures of pacemakers and defibrillators,^18^, ^19^, ^20^ including biventricular devices^21^, ^22^; such systems are especially required as an alternative to fluoroscopy in pacing procedures involving pregnant women and children.^23^, ^24^

In 2017, the first case of HBP accomplished with the help of NFM was reported; the Authors used an electrophysiological mapping catheter introduced via a femoral venous approach to create a 3D geometry of the cardiac veins and chambers.^25^ Subsequently, other case reports or series of HBP performed with the help of NFM were published.^26^, ^27^, ^28^, ^29^, ^30^, ^31^, ^32^ In these papers, a striking reduction in FT was always reported, without compromising the effectiveness of the procedure or increasing procedure‐related complications. The 3D right heart geometry anatomical map was created in an additional step by means of an electrophysiological catheter, introduced via the femoral or the axillary vein, with the exception of Richter et al, who acquired the 3D map directly by means of the HBP catheter. Accordingly, in comparison to a conventional implant control group, not only FT but also PT was reduced in this study, although not significantly.^30^ Conversely, Sharma et al in their case series reported a longer PT in the NFM subgroup than in the conventional implant subgroup (134 vs 100 min) by performing the anatomical 3D map by a separate electrophysiological catheter.^28^

The novel NFM system KODEX‐EPD reconstructs high‐resolution images of cardiac anatomy based on the distinct dielectric properties of biological tissues, by inducing anisotropic electrical fields within the patients' body and measuring the resultant electrical field differences on the catheter electrodes, as they move in the heart chambers. The anatomy of cardiac structures and veins can also be acquired at a short distance around the catheter electrodes (even without physical contact with the endocardial surface) and displayed not only as a 3D map but also as a virtual bidimensional panoramic image, which allows all the anatomically relevant structures to be seen in one view. Importantly, the system can work with any type of electrophysiological or pacing catheter, that can be directly navigated on the map.^10^ Previous studies showed that the image quality generated by the KODEX‐EPD was noninferior to other currently used NFM systems.^10^, ^33^

The KODEX‐EPD system has been successfully used in catheter ablation as well as in pacing procedures.^34^, ^35^ It was first used in permanent HBP by Hua et al^36^; in this paper, the pacing lead itself was used to acquire a right atrial map and localize the His bundle region; similarly, another lead was placed in the right atrial appendage without fluoroscopy. Subsequently, the same group presented a series of 10 patients with atrioventricular block or sinus node dysfunction who underwent HBP with the use of the KODEX‐EPD system, aiming to compare procedural outcomes with those of a control group who achieved HBP using the conventional fluoroscopic approach^37^; however, in this study, the anatomical map was performed in a separate step by using a roving quadripolar catheter. The study results showed that FT was strikingly reduced in the Kodex‐guided group (1.45 ± 0.58 vs 12.36 ± 5.46 min, *p* < .01) whereas PT was not (85.40 ± 22.34 vs 86.50 ± 15.05 min, *p* = .90).

We performed HBP in a population of patients with mixed indications (CRT in 26% of cases), by using two different pacing catheters: the widely used Medtronic 3830 and the Biotronik Solia, the latter having recently been adopted for HBP purposes; the procedures were performed by several operators, some of whom were at the beginning of their learning curve. All these elements could explain why we obtained a FT of 3.03 min in the treatment group, which is relatively high compared to other studies. On the contrary, PT both in the treatment and in the control group was the shortest ever reported among studies that included a control group. This, in our opinion, underlines the usefulness of building the anatomical map with the pacing catheter instead of using an additional catheter, in order to reduce the duration of the procedure, as previously reported by Richter et al.^30^

## 5 CONCLUSIONS

Our study confirms the feasibility and safety of using the KODEX‐EPD system in HBP procedures. Moreover, the KODEX‐EPD system is effective in markedly reducing procedural radiological exposure, as evidenced by the significant drop in FT; PT has not increased, in contrast, provided that the anatomical map is directly constructed using the pacing catheter.

## 6 LIMITATIONS

The main limitation of this study consists of its nonrandomized nature; furthermore, a relatively small number of patients were included.

As a result of the multicenter design of the study, different operators performed the procedures; they were at different stages of their learning curve, while a more accurate evaluation of the advantages conferred using a NFM system would have required all operators to complete the learning curve.

Radiation exposure in our study was only quantified by FT; we did not estimate dose‐area products, which is the most widely used method of skin dose estimation. However, for electrophysiological procedures the relation between skin dose and FT was found to be good.

The present study has not been designed to evaluate if the use of different catheters or stylet can lead to different outcomes; future studies, with different designs, will be produced to answer this question.

Finally, FT and PT were calculated for the entire procedure; no specific information about any procedural step (i.e. HBP procedure) has been appointed.

## CONFLICT OF INTEREST

Authors declare no conflict of interests for this article.
